# Excerpta

**Published:** 1880-11

**Authors:** 


### EXCERPTA.

### CAPTAIN EADS' SHIP RAILWAY.

The *Scientific American* of this week contains two full page illustrations of Captain Eads' proposed railway for transporting ships with their cargo across continents.

Captain Eads claims by his plan to be able to take loaded ships of the largest tonnage from one ocean to the other across the Isthmus of Panama, as readily as can be done by a canal after the Lessep plan, and at a much less cost for engineering construction.

The project is certainly bold and ingenious, and the projector anticipates no serious difficulties in carrying forward his enterprise. The engravings referred to in the *Scientific American* show the proposed construction of not only the railroad, but the appliances for transferring the ships from the water to the rail.

In addition to the large number of engravings, illustrative of engineering works, inventions and new discoveries which appear weekly, the *Scientific American* has, during the past year, devoted considerable space to illustrating and describing leading establishments devoted to different manufacturing industries.

This feature has added very much to the attractiveness and usefulness of the paper. More than fifty of the most important industrial establishments of our country have been illustrated, and the processes of the different manufactures described in its colums. The *Scientific American* has been published for more than thirty-four years by Munn & Co., 37 Park Row, N. Y., and has attained a larger weekly circulation than all similar papers published in the country. The publishers assure the public that they have not printed less than 50,000 copies a week for several months.

### LEARNING TO SMOKE.

Parents should be on their guard and endeavor to prevent their boys learning to smoke. The habit is now usually acquired in boyhood, and many boys are addicted to it.

An Englishman, fond of smoking, said once to Neal Dow:—" Men never acquire the habit, or very rarely, and then under exceptional circumstances. It's boys who learn, because they think it manly to use tobacco. They steal away into secret places ; they hide behind the barn or creep under the wood shed, out of sight, because they are ashamed, and there they smoke and vomit. That's the way in which ninety-nine of every hundred tobacco users have acquired the habit."

As to the effects of tobacco on pupils or students, Yale College furnishes the following statistics:—

" Each class is graded in divisions according to scholarship, the best scholars being in the first, and so on down to the fourth, where they are, in the slang of the campus, 'not too good' but 'just good enough' to keep hanging by the eyelids. In the Junior Class it was found that only 10 out of 40 in the first division were addicted to smoking ; 18 out of 37 in the second ; 20 out of 27 in the third ; and 22 out of 26 in the fourth. The proportion of smokers, it will be observed, increases in regular ratio with the falling off in scholarship." In reference to this, it may be that dull or stupid boys are more disposed to smoking.

Amaurosis (loss of sight,) and heart disease are among the most common effects of using tobacco, especially in young persons.

*Canada Health Journal.*

### INFLUENCE OF ELECTRICITY ON BACTERIA.

Some new experiments with regard to the influence of electricity on bacteria have been published by Professor Cohn, who adopted the method of sowing with bacteria a sterilised mineral nutritive solution, subjecting them to electric currents, and noting the results. A Marie Davy flask element he finds to exert (according to strength of current,) either no influence on the increase of bacteria, or merely a retardative influence. On the other hand, the current from two powerful elements sterilised the nutritive solution completely at the positive pole in twelve to twenty-four hours, so that afterwards the bacteria introduced did not increase. At the negative pole, the action was weaker, the liquid not completely sterilised. At neither of the poles were the bacteria killed, and when brought into another nutritive liquid they developed normally ; on the other hand, yeast cells, and mycelium fungus brought into the liquid that was sterile for bacteria, increased plentifully at the positive pole. A battery of five strong elements killed the bacteria distributed in the liquid within twenty-four hours, and sterilised the liquid in both poles.

*Med. Press and Cicular.*

### ON THE PREVENTION OF NEAR-SIGHT IN THE YOUNG.

BY HASKET DERBY, M. D.

A boy or girl is observed by the teacher, during the early days of school life, to see maps and drawings on the blackboard, across the room, less readily than the other scholars. After a time the parents' notice may be called to the fact, and in a small number of cases the child is brought to the surgeon. Expressions of incredulity are subsequently exchanged for those of astonishment when it is demonstrated to the parents that the child cannot see the largest letter of the test card across the room, and that his farthest point of accurate vision is perhaps within twelve inches of his eyes. He has, without the knowledge of his family, become near-sighted. A difficulty has been fastened upon him that will act to his serious disadvantage through life, and the tendency to which he may transmit to his children. Though its progress may be modified by following suitable advice, it is now no longer capable of removal. Such cases occur with great frequency.

According to the best attainable statistics, there is found in the United States only about one-third as much near-sight as is met with in Europe. And yet its prevalence in New England may be estimated from the fact that one person in ten who consults the ophthalmic surgeon does so on account of this very difficulty.

That near-sightedness is always a disadvantage to its possessor, that such an eye is in fact a diseased eye, that the affection is one that tends between certain ages to increase, and that exceptional increase may with advancing years lead to blindness, are facts so often insisted on as to have become familiar to all who take an interest in this subject. Few states of the eye have been more accurately studied. The statistics collected have reached the limit of usefulness. The age at which myopia is likely to begin, the period of life during which it generally increases, the influence of civilization and education on its development, are all satisfactorily known. The hopelessness of its cure is universally conceded. But, in my opinion, far too little study has been bestowed on the possibility of its prevention. It is to this branch of the subject that I briefly invite attention.

The first question that arises, then, is as to whether prevention is possible. In answering this, two facts are to be taken into consideration: 1st. that near-sight is seldom, if ever, congenital, though a tendency to its development may undoubtedly be inherited. 2d. that it is usually a product of civilization. This view receives support from those who have had the opportunity of examining the eyes of savage tribes. Furnari, among others, found no near-sight among the Kabyls. Macnamara states that he, some years ago, was among the Southals, the aborigines of Bengal, dwelling among the Rajahmahal hills. "I took," he says, "every opportunity of examining the eyes of the people I was brought in contact with, for the purpose of discovering if myopia and such like diseases existed among them, but I never yet saw a young Southal whose eyes were not emmetropic," (that is, perfect.) Other testimony is in the same direction.

The following is a brief summary of our knowledge regarding the appearance and progress of near-sight:

It is not generally found at all among children who have not commenced school life.

Between the ages of six and seven, some three school-children in a hundred are found, in this country, to be near-sighted.

This percentage steadily increases, and at the age of twenty at least twenty-six in a hundred are thus affected. The percentage rises to forty-two in Russia and sixty-two in Germany.

Other things being equal, the children of near-sighted parents are more apt to acquire near-sight than are those whose parents have normal vision. The development of near-sight is furthered by the following causes:

1. Work by insufficient light.
2. Work on minute objects, such as fine print, intricate maps, and the like.
3. Work in a constrained or stooping position.
4. Continuous study, and
5. Prolonged or excessive study.

The action of this last is well illustrated by an example given by

Erismann. Four thousand three hundred and seventy-eight scholars being in the habit of studying out of the regular school hours, he found

Of those studying two extra hours 17 per cent. were near-sighted.

Of those studying four extra hours 29 per cent. were near-sighted.

Of those studying six extra hours 40 per cent. were near-sighted.

Such being the causes of near-sight, a large portion of the success that is likely to attend its prevention would depend on their judicious elimination. So much has already been written on this subject that it is needless to reiterate the advice given from every quarter. The necessity of well-lighted rooms, of clearly-printed text books, of properly constructed desks and seats, has been clearly dwelt upon. The subject of prolonged study must be left to those more familiar than myself with the forcing system of modern education. A single word may, however, be permitted regarding the continuous use of the eyes in young children, and the mechanism of the production of near-sight.

A normal eye becomes myopic, of course, by growing longer, by the bulging out of its posterior segment. There is excellent authority for believing that this is peculiarly apt to take place in children of near-sighted parents. They inherit a diminished power of resistance in this part of the organ of sight. The length of the eye may be normal at birth, but the scleral tissue of its posterior half is unduly elastic, and gives way more readily than it ought during the period of the development of the body. After this time is passed, it may acquire new strength, and cease, after the twentieth year, to be liable to give way. Let us bear this tendency in mind, and remember, moreover, Eulenburg's statement that ninety per cent. of curvatures of the spine, which do not arise from a special disease, are developed during school life. If now, in the case of a growing child, a constrained and unnatural position, long continued, may cause malformation of so solid a structure as the vertebral column, why may not a similarly constrained position (so to speak) of the eye cause a change in the shape of that organ? A bow must be kept unstrung to retain its elasticity. The continued effort of accommodation for near objects is accomplished by the change of shape of the crystalline lens from its least to its greatest convexity. And just as the bow, too long or injudiciously stretched, fails to regain its pristine shape, so does the crystalline, overstrained or fatigued, often refuse for a greater or less time to relax into the shape of rest, when the eye is sought to be adapted for only distant objects. This inability to relax we call "cramp of the ciliary muscle," "spasm of the accommodation," and it is the first step in a downward course.

Now one of the principal factors in bringing about the change of shape or lengthening in the posterior segment of the globe is this very exercise of the cccommodation. It probably acts by throwing the choroid into a state of tention and inducing congestion. The eyes, therefore, used so continuously as to undergo spasms of the accommodation, are even more likely to become near-sighted than those in which the accommodation is relaxed as soon as near objects are no longer regarded.

Near-sight may then begin with spasm of the accommodation. A single case will serve as an illustration of this fact.

I examined the eyes of a student at Amherst College, at the commencement of his freshman year, in November, 1875. He presented himself toward the close of the day; the afternoon was a dark one, and he had just been reciting. There was a slight degree of near-sight in each eye. In October of the next year I examined him again, and found his near-sight entirely gone. He had been laboring under accommodative spasm. But in June, 1879, at the completion of his college course, true near-sight, to a considerable amount, had made its appearance, and the ophthalmoscope showed it to be real, and not due to spasm.

This case is of great interest as illustrating the theory that near-sight frequently commences in the above manner; how frequently cannot at present be estimated. According to many, such cases are the rule; others think them the exception. But allow the fact that they do exist, and we have an important clew to the prevention of near-sight; for this spasm or cramp of the ciliary muscle yields very readily to treatment. How often do we hear complaints from children that, after studying a time, distant objects appear blurred and and indistinct! They can no longer tell time by the clock across the room, no longer make out figures on the blackboard. Such cases are often brought to the opthalmic surgeon. With some of the children the spasm has already disappeared. With others a rest of a few days suffices to remove it. Others, again, require to be put under the full influence of atropine, to which, sooner or later, the thing must yield, as in the following instance:

E. S., at the age of 12, had never considered himself near-sighted. Both parents have normal vision. His brother, aged seventeen, who came under my care about the same time, had a considerable amount of myopia, which up to the present has gone on steadily increasing. His sister, slightly older than himself, is similarly affected. In March of this year E. S. came to me again, complaining of pain in eyes on use, and inability to see distant objects distinctly. He was now near-sighted, and could not see sharply any object removed more than thirty-nine inches from him. I kept him three weeks under atropine, and the near-sight entirely disappeared, he being able, according to his own expression, to see as far as ever. We are warranted in the supposition that, had the difficulty not been recognized and the treatment employed, he would, like his brother and sister, have become near-sighted for life.

Assuming, then, as we are amply justified in doing, that cramp of the ciliary muscle is the first step in the development of near-sight in a number of cases, and knowing that the spasm, as such, is readily curable, the desirability of watching for its occurrence becomes evident. In, fact, the extreme importance of recognizing all near-sight in its very earliest stage has been far too little insisted upon, for it is at this precise period that so much may be expected from judicious treatment, as well as from careful regulation of the child's habits, method of study, and length of time spent in school.

Unless measures be taken to prevent it, the percentage of near-sight among us is liable to increase as time goes on, largely through the transmission of the hereditary tendency.

What course shall be adopted to prevent this? I would suggest the following plan: Let all children have their vision tested as soon as they know their letters; let a careful record be kept of the result; have the examination repeated at least twice a year during the whole of school life; and let near-sight be promptly treated as soon as its commencement is detected.

The school committee of Boston, for instance, might adopt some such plan as the following: A brief document, couched in popular terms, could be circulated among the teachers of the public schools, explaining the views just rehearsed, and giving instructions in the use of the test-types of Snellen or Monoyer. Each school should be provided with one or both of these cards, the price of which is trifling. The card itself should be hung in a room of sufficient length, and illuminated artificially at the time of examination, thus securing uniform intensity of light on every occasion. At its entrance into the school each child should have its vision tested by the card, in each eye separately, and a record kept of the result. This examination might be repeated at least once in six months during school life, and any change noted.

Thus far the whole matter may be left in the hands of a tolerably instructed teacher. If, now, a child that has previously had perfect sight is observed to see less and less on successive trials, the assumption is that near-sight is commencing. The family should now at once be notified and advised to apply to their physician. With the instruction now given in our leading medical schools, it will be easy for him to carry the examination further, to recognize the presence of near-sight, and to prescribe the necessary rest or atropine treatment, that, if the case has been taken in time, will restore the eye to its normal condition. If the myopia refuses to yield to antropine, and in moderate amount has to be accepted as a life-long disadvantage, suitable advice will certainly modify its progress.

If sufficient interest can only be awakened in the subject, and if this course be followed, I do not see why the amount of near-sight in the community may not ultimately be considerably diminished. Let the absurd traditions of the past, that myopia is due to an increased convexity of the eye, and is even an evidence of strength of sight, be once fair banished from the popular mind. Let it be insisted on in and out of season that near-sight is a disease, a product of civilization, arising generally during school life, often curable if promptly met, always incurable and even progressive if neglected, invariably a disdvantage to its victim, and sometimes in later life fatal to sight; let these facts be fairly brought forward and understood, and the next generation will have better and stronger eyes than those of the past, and that of the present.—*Boston Med. and Surg. Journal.*

### CÆSAREAN SECTION ON A DWARF.

The operation of Cæsarean section was successfully performed upon a dwarf, Mrs. Wm. Burnell, in Philadelphia, recently. The infant was doing well, and the mother was in a fair way to recover at last accounts. The dwarf is thirty-two years old, and forty-two inches high. Her father was a dwarf, about the same size as herself, while her mother, who died when she was a baby, was a woman of ordinary size. About nine months ago she married William Burnell, a negro minstrel, then connected with the same show. Mr. Burnell is not a dwarf, but a full-grown man. Such marriages are unequal to the last degree, and ought not to be allowed in civilized and presumably intelligent communities.—*Medical Record.*

### TO BLEACH DISCOLORED TEETH.

J. P. HOLMES, MACON, GA.

After treating and curing the abscess on the root of the tooth, if it has one, the first thing to be done is to thoroughly clean and scrape well the entire inside of the tooth from the point of root to the orifice of the cavity. This must be well and thoroughly done, removing all decay and softened bone structure and the stain as far as possible. This having been perfectly done, the root must be filled just to the nerve chamber or just below the line of the gum. Our practice is to moisten the first piece of gold with carbolic acid, carry it as near to the apex of the root as possible, then fill the balance with gold to the line mentioned. We now take *fresh* chloride of lime and chloroform, mix and make a thin paste with them, after which we fill the cavity full, leaving only a small space near the out edge or cavity for a gutta percha stopping which we stop cavity with. The patient is dismissed until the next day or the one following, when the stopping and chloride of lime is removed and a fresh lot is inserted as before. This is repeated for two or three sittings, when the tooth must be thoroughly cleaned out to the gold filling in the root, wiped as dry as possible and the entire cavity filled with the *whitest* oxy chloride zinc made. As soon as filled an old instrument is heated and applied to the filling. This is repeated until it is quite hard, which will only require a few moments ; as soon as made hard with an excavator or one with the engine, cut out and shape the cavity to suit, then with gold which must be prepared before you commence the operation, you fill as usual. After filling and finishing the filling, make a paste with glycerine and pumice stone, apply to the tooth on a corrugated rubber point with the engine, cleaning it as thoroughly as you can. When this is done the tooth will look as white as it can be made by any means.—*Dental Lumiuary.*

### MERCURIAL AMALGAMS.

BY M. G. CUNNINGHAM.

After twenty-five years of stubborn fight supporters of gold as a filling for decayed teeth accept the possibility of plastic material being in certain cases its superior; throughout this period I have been content to hold my peace and act entirely on my own judgment in the selection of material, as however, it seems to be the fact that a man who uses plastic filling without danger of being termed a "quack," may speak, I would, through your kind agency, convey to brother Dentists my method of preparing metallic amalgams, which has saved me much trouble and my patients a large number of teeth.

In using amalgam, the first thing we ought to take into consideration is whether that which we are using and calling by that name is such, and I ventnre to say that in a very large number of cases it is no amalgam at all, but a concrete admixture of solid metals with liquid mercury. In the early days, when metallic precipitate of silver was employed, perfect amalgamation was not difficult to obtain, provided the precipitate had been in the first instance properly washed and carefully stoppered, the minute sub-division of the metal and absence of oxidation aiding largely to this result. The fillings of the present day are of a totally different character, coarse in grain, and of a nature to oxydize on even momentary contact with air, they become difficult to amalgamate with mercury, which, in itself, is a highly oxidizable metal, so that recourse is often had to a glass tube and violent agitation to produce that which is at best only a semblance of what it should be—a thoroughly homogeneous mass, that upon setting will retain a uniform texture and density proportionate to the constituents of which the fillings are composed. If, however, to the fillings be added a drop or less of sulphuric acid, either in the palm of the hand or mortar, it will be found that the metals will almost instantaneously amalgamate, whilst the oxides combining with the acid leave a residuum which, by its quantity, clearly shows what a very imperfect body could have been a so-called amalgam containing only a small portion of them. Washing in pure water at once removes all trace of acid, and a thoroughly reliable stopping can be at once produced from materials otherwise worse than useless.

—*Monthly Review of Dental Surgery.*

### A POSITIVE SIGN OF PREGNANCY DURING THE FIRST THREE MONTHS.

In a paper read before the Detroit Academy of Medicine, Dr. J. H. Carstens mentioned a positive sign of pregnancy on which he had always relied, and which had never failed in his experience to enable him to diagnosticate this condition. He had been under the impression that it was a new, not hitherto described sign; but looking over the literature of the subject, he found that it had been mentioned years ago by Jacquemier and others. It seemed, however, to have fallen into oblivion, and was not mentioned in the text-books. He referred to the color of the mucous membrane of the vagina and cervix uteri. This he always found of a purplish blue, or rather deep violet hue, in pregnant women, and he depended on this peculiar color in making a diagnosis of pregnancy in the first, second and third months. He said that it had never failed, and that it was not produced by any pathological condition. The different colors produced by uterine diseases could not be mistaken for this pathognomonic violet hue. He had often called the attention of students to this sign. It had been claimed by some that this color of the mucous membrane was found in various pathological states. He claimed that the discoloration in such cases was different from that found during pregnancy. It was more blue and scarlet, mixed or motled, nor was the peculiar soft, velvety condition of the membrane present. He simply called it violet, and said that it must be seen, and then it would never be forgotten. It was probably caused by engorgement of the veins. All he asked was that this sign be again looked for and submitted to a rigid investigation, and he was sure the verdict would be that it was the only sure sign we had at present to diagnosticate pregnancy from the first few weeks up to the fourth month.—*Detroit Lancet.*

### PAINLESS OPERATION FOR INGROWING NAILS.

BY J. H. CONVERSE, M. D.

I will give this foı the benefit of your readers, as perhaps it will be new to some of them. It consists of wedging cotton under the free margin of the nail, placing over it a piece of adhesive plaster with a hole cut into it the size and shape of nail to be removed ; then moisten the end of a pencil of caustic silver and apply it to part to be removed, taking care not to touch any other portion. The next day the nail will have assumed a black or brown appearance. Upon raising the nail it will be found to have become separated from the subadjacent tissue, and all there is required to complete the cure is to clip off the dead portion.—*American Medical Journal.*

IODIDE OF ETHYL AND PILOCARPIN IN ASTHMA.—Iodide ot ethyl has been used successfully by Dr. D. R. Brower (*Chic. Med. Jour. and Ex.*), after all other remedies had failed. The patient was a boy of fifteen, with an inherited nervous weakness. The dose employed was six drops. The drug relieved the paroxysms and lessened the intervals between them. Dr. Burkhart finds pilocarpin curative in asthmatic cases complicated with chronic bronchitis.

### BEEF TEA AND ITS VALUE.

Certain iatro-chemist and iatro-physiologists, not being able to discover what were the nutrient elements in beef extract and beef tea, have of late years much decried those preparations. At most, they allowed them to be "condiments." Fortunately, common sense and clinical experience are gaining the day over the theoreticians, and we take pleasure in publishing the following details contributed by Mr. Wilkinson, House-Governor of St. Mary's Hospital, London, to the *British Medical Journal*:—

The mode of preparing beef tea at St. Mary's Hospital is as follows : The meat is cut into small pieces, and placed, in the evening, in an earthenware vessel, with sufficient cold water to cover the meat ; in this it is allowed to remain all night. In the morning the meat is taken out, placed in other water, and boiled for several hours. The meat of the previous day is then passed through a mincing machine, and put into the cold liquor in which the meat was steeped the previous night, and upon this the boiling liquor from the day's beef tea is poured, and the whole well stirred, and it then forms the complete beef-tea. The characteristics of good beef tea are, that all the nutritious elements of the beef should be made available; and by the process carried out as above, this is effectually done, the albumen, fibrine, and gelatine being all retained and taken by the patient. Moreover, by the above method a much smaller quantity of meat is required than under the ordinary mode, and it would, consequently, not become a jelly if allowed to stand; but by adding a larger qnantity of beef this result could, of course, be obtained. (This forms with us what is called beef jelly.) It should, however, be remarked that in very hot weather the beef tea cannot be made in this manner, as it would become sour, from the length of time required for its preparation.

### SHOULD WE BANDAGE AFTER LABOR, AND WHY? AND IF NOT, WHY NOT?

BY ALEXANDER ELWELL, M. D.

These questions have presented themselves to my mind, from the fact that I have found a growing disposition on the part of some of my brethren to break in upon the long and useful custom of bandaging our parturient patients.

Not long since, I called to my assistance, in a case of labor, a gentleman whom I have always held in high esteem, both for his medical opinion as well as his gentlemanly and social qualities, who, upon the question about the bandage, remarked that it was of but little importance whether she was bandaged or not; this set me to thinking over the subject, a subject that Prof. Hodge in his lectures laid much stress upon, and which other authors, such as Dewees, Meigs, James and Ramsbotham, had led me to think *was* of very much importance. The disciples of Hannheman do not bandage, but I see no cause why we should follow in their footsteps. The principle object which the bandage serves is to brace the bowels, and give artificial snpport in the place of that which they have lost through the extreme laxity of the abdominal muscles, and to prevent the faintiness frequently attendant on the sudden removal of certain degree of pressure. I also think that a bandage, properly applied, stimulates the uterus to contraction; though too much reliance must not be placed on it for that purpose, but the uterus should be induced to contraction before the application of the bandage by gentle and continued friction over and to it. Again, I think the bandage serves other purposes—such as the prevention of anteversion, lateral obliquities, and even prolapsis—by relieving uterus of the weight ot over-distended muscles and *dislocated intestines*, until nature and time comes to the relief, giving tone and strength to surroundings. My idea of a bandage is one that is wide enough to come from the ensiform cartilage, well down over the hips, with gussets or gores, fitting smoothly, showing but little disposition to work up; it should be worn from six to eight weeks; then we will find our patients to walk with more erect, firm, and elastic step, complaining less with back-ache and head-ache and general lassitude. A bandage thus made and worn, with the addition of two or three napkins under it, next to the skin, will keep us advised of any tendency to hemorrhage, should it exist.

I am certain I have seen much flooding where there was no importance attached to bandaging, or none applied, that might have been avoided in part, or perhaps altogether. Sometimes, in hasty delivery, the birth occurring before the arrival of the accoucher, the patient being allowed to lie without the proper attention, concealed hemorrhage does occur. One such case comes very vividly to my mind. I was sent for, and upon my arrival I found, to my astonishment, my patient dead; the child was born, and the placenta self-delivered. Upon questioning those around, no satisfactory answer could I get as to the supposed cause of this sudden taking off. Had there been much flooding? I asked. Not more than usual in cases of confinement. I was not satisfied, and made an examination post mort, and found the uterus filled with clotted blood, and as large as the seventh month of pregnancy. Now, had the uterus been held firmly in place by bandage and napkins, I cannot help thinking that much of the flooding might have been avoided, and the patients' life saved. Other similar cases I have seen, and am led to believe that the timely use of the bandage, and preceeding manipulations, saved my patient. So thoroughly am I convinced of their usefulness, I would not dare to leave off their use.

I have endeavored to give some reasons why the bandage should be used, and the negative side of the question I leave for those to answer that may differ from me. Should there be good reason given why bandaging may or should be done away with, then would I consider we were rid of very much trouble.

Miss Roberts, of Steuben county, New York, was engaged to be married to a gentleman of Maryland. She had recently had a set of artificial teeth made by a dentist of that place, and while she was visiting Maryland last week the dentist called on her, and asked how her teeth were lasting. She handed them to him, and he put them in his pocket, saying "You can have these teeth when you pay for them." Miss Roberts was not able to pay for them just then, and the dentist carried them away. That night the man who promised to make Miss Roberts his wife called to see her, and she sent word down that she could not see him that evening. He insisted upon an explanation, and Miss Roberts' friends explained. The gentleman went away. Next day he wrote to Miss Roberts that he did not know she wore artificial teeth, and that he could not marry a woman who wore them. Miss Roberts fancies that she can recover $5,000 from the dentist for the loss a husband, and for annoyance growing out of his taking the teeth from her, and moreover, that she can recover damages from her late suitor, in a breach of promise case.

—*Dental Advertiser.*

VALUE OF METHOD.—Method in everything is incalculable. It promotes comfort—it saves a large expenditure of time—it avoids numberless inconveniences—it is of great moment in relation to mind and character; and it is essential to the dispatch of all business; for that which is well arranged proceeds with ease and regularity.

A Doctor in Scotland made a nerve and bone all-healing salve and thought he would experiment a little with it. He first cut off his dog's tail and applied some of the salve to the stump. A new tail grew out immediately. He then applied some to the tail which he cut off, and a new dog grew out. He did not know which dog was which.

